# Attitudes Toward Money and Control Strategies of Financial Behavior: A Comparison Between Overindebted and Non-overindebted Consumers

**DOI:** 10.3389/fpsyg.2021.566594

**Published:** 2021-04-16

**Authors:** Filipa de Almeida, Mário B. Ferreira, Jerônimo C. Soro, Carla Sofia Silva

**Affiliations:** ^1^Universidade Católica Portuguesa, Católica Lisbon School of Business and Economics, Lisbon, Portugal; ^2^Faculdade de Psicologia, Universidade de Lisboa, Lisbon, Portugal; ^3^CICPSI, Faculdade de Psicologia, Universidade de Lisboa, Lisbon, Portugal

**Keywords:** overindebtedness, financial behavior, money attitudes, debt, financial management

## Abstract

This paper addresses whether overindebted and non-overindebted consumers differ in their attitude toward money (specifically, the degree to which consumers care about money and feel difficulties keeping track of their money) and how this attitude impacts three different financial behavior categories: record keeping (e.g., recording spending in writing), adjusting balance (e.g., trying to find ways to decrease one’s expenses to match income), and monitoring balance (e.g., monitoring one’s spending to see if it is in line with what is expected). Overindebted consumers were recruited via an NGO for consumer defense and were categorized (whenever possible) into two subgroups: consumers who became overindebted due to internal causes (e.g., bad financial management) and consumers who became overindebted due to external causes (e.g., unemployment). Non-overindebted consumers were a convenience sample. Non-overindebted consumers showed more positive attitudes toward money than both groups of overindebted consumers and overindebted due to external causes showed more positive attitudes than overindebted consumers due to internal causes. All groups share similar financial management behaviors except for monitoring balance, which was more frequent among non-overindebted consumers. Furthermore, a regression analysis indicates that money attitudes helped explain financial behavior differences between consumers above and beyond their indebtedness status. Consumers’ attitude predicted financial behaviors, even when controlling for relevant socioeconomic variables (education, income, age, and gender). Further analyses comparing money attitudes and financial behavior for the three subgroups (non-overindebted, overindebted due to internal causes, and overindebted due to external causes) showed no differences.

## Introduction

Contracting debt facilitates consumption and investments, contributing to growth and stability at a macro-economic level (Ando and Modigliani, 1963; Modigliani, 1966; Fan et al., 1993; Hanna et al., 1995; Cecchetti et al., 2011). However, debt becomes worrisome to individuals and governments alike when it reaches such high levels that households become overindebted, that is, they begin to face recurrent hardships and are unable to meet their financial commitments (Haas, 2006; D’Alessio and Iezzi, 2013).

Prior research has clearly shown that overindebtedness is a serious social problem with long term negative consequences for households’ life, including poorer health, anxiety and decreased well-being (Norvilitis et al., 2003; O’Neill et al., 2006), self-control lapses (Peltier et al., 2016), financial stress (Xiao et al., 2006), increased feelings of failure (Robb and Pinto, 2010), family conflict (Bloom et al., 1985; Kerkmann et al., 2000), and divorce (Dew, 2011). Unfortunately, household indebtedness levels have been rising since the 2008 economic World crisis (Bover et al., 2016), with households carrying debt close to (and into) retirement (Lusardi et al., 2018).

Among the common culprits of overindebtedness are external factors that include unexpected life events (e.g., unemployment; Canner et al., 1991; Niemi-Kiesiläinen, 2009), lack of acquired skills, such as financial literacy (Norvilitis et al., 2006; Robb, 2011; Lusardi, 2012, 2019; Lusardi and Tufano, 2015) often coupled with judgment biases stemming from heuristics (e.g., Thaler and Sunstein, 2008). Similarly, internal or individualistic factors, such as impulsivity and low self-control (Baumeister et al., 2007) put consumers at higher risk of overindebtedness (Ameriks et al., 2003, 2007). Other research has suggested that lack of self-control is more a consequence than a cause of financial scarcity and overindebtedness. According to this account, being mentally preoccupied with making ends meet leads to cognitive depletion and emotional distress, which negatively impacts decision making, thereby leading to impulsive financial choices (Mani et al., 2013). Given the large number of factores associated with overindebtedness (some times as causes other times as effects) it follows that the specific reasons why a household becomes overindebted must be considered for a comprehensive aproach of this problem (e.g., Ferreira et al., 2020).

This paper aims to contribute to better understanding the risk factors of overindebtedness by focusing on a relatively less investigated potential cause, namely consumers’ attitudes toward money (how much they care and keep track of their money) and three different categories of money management behavior (record keeping, adjusting balance, and monitoring balance). Moreover, in contrast to most studies, which usually assess how consumers’ attitudes impact debt in general, in this paper the attitudes and financial management behaviors of both overindebted and non-overindebted consumers are studied. This is a relevant categorization since amount of debt is not tantamount to being overindebted. Overindebtedness depends on the ratio between income and loan repayments.

Finally, in order to look for differences among distinct types of overindebtedness, the overindebted households who participated in our study were categorized (based on self-reported causes of indebtedness) in cases of overindebtedness that resulted from internal causes (e.g., bad financial management; low self-control) and cases that resulted from external causes (e.g., unemployment, salary cuts, sudden death in the family).

### Money Attitudes

Different measures of money atttitudes have been developed through out the years. Yamauchi and Templer (1982) developed the first well-known money attitudes scale (MAS). Other important measures of money attitudes include Furnham’s (1984) Money Beliefs and Behavior Scale and Tang’s (1992) money ethic scale. Although with somewhat different factorial structures, which have evolved as more research with these and other attitude measures was done (e.g., Tang, 1993, 1995; Roberts and Sepulveda, 1999; Klontz et al., 2011; Lay and Furnham, 2019), the most important dimensions that emerged from the different attitudes scales can be described in terms of the cognitive, affective, and behavioral components of the tripartite model of attitudes first proposed by Rosenberg and Hovland (1960; see also, Eagly and Chaiken, 1993; Chatterjee et al., 2018). For instance, the main dimensions of the MAS include (a) the perception of money as a source of freedom/power and achievement (cognitive component); (b) The association of money with financial planning and budget (behavior component); and (c) money as a source of distrust, suspicious and anxiety as well as protection from anxiety (affective component).

Since one of our main goals is to evaluate how money attitudes impact financial behaviors, the measure used herein focus on the behavioral component of these attitudes (i.e., how much consumers take care of and monitor their money) as the factor that may influence one or more of the three categories of financial behavior investigated. Indeed, although Rosenberg and Hovland (1960; see also Kaiser and Wilson, 2019) argued that the three components of attitudes correspond to manifestations of the same latent variable (i.e., attitudes toward money), the attitude’s behavioral component is likely to be a more direct indicator of related financial behaviors than the cognitive and affective components.

### Money Attitudes, Financial Management Behavior, and Debt

Prior research on money attitudes suggests individuals demonstrate a variety of predisposed responses toward money. Money has different meanings and serves different purposes for different individuals, leading them to act differently toward it. Importantly for this research, attitudes regarding one’s actions toward money vary considerably as well, with some demonstrating ease in spending and accruing debt and others seeming more anxious and more devoted to saving money.

Money attitudes have been shown to have a significant positive impact on financial management behavior among young adults (Qamar et al., 2016). In their study, Qamar et al. used a money attitude questionnaire that included measures of money avoidance, worship, status and vigilance (Klontz et al., 2011) and found that 20.9% of the personal financial management behavior was explained by money attitudes.

Dowling et al. (2009) showed that financial management practices and money attitudes – measured in terms of (a) the importance ascribed to the ownership of material goods, (b) the extent to which one uses money as a standard of comparison with others; and (c) the extent to which individuals think and worry about money – predicted financial problems among young male Australian workers.

Similarly, Klontz and Britt (2012) showed that core attitudes and beliefs about money drive financial behaviors (see also Kahler and Fox, 2005). In their study three belief patterns (money avoidance, money status, and money worship) were not only associated with lower levels of total assets, lower earnings, and higher measures of revolving credit but could also foresee disordered money practices such as impulsive purchasing and financial dependence. In contrast, attitudes and convictions favoring money vigilance, including frugality, caution, and anxiety about money, appeared to be protective against poor finances and dangerous financial practices.

More recently, Sabri et al. (2020), studied the determinants of employees’ financial well-being in Malaysia and found out that money attitudes, substantially contributed to employee’s fulfilment of current and ongoing financial obligations (above and beyond financial practices, self-efficacy, and emotional coping).

Money attitudes have also been shown to be a predictor of credit card debt. Specifically, the money attitude of effort/ability appears to play an important role (together with attitude toward credit) in distinguishing between more indebted students (with four or more credit cards) and less indebted ones (with one to three credit cards; Hayhoe et al., 1999). Likewise, good financial management practices (e.g., budgeting, saving, and regulating spending; see Godwin and Koonce, 1992) were also shown to be a main predictor of debt levels (Lea et al., 1995; Donnelly et al., 2012; Ksendzova et al., 2017).

Taken together, these studies provide strong evidence concerning the impact of money attitudes on consumers’ financial behavior and show that both attitudes and financial behaviors are important predictors of indebtedness. However, in contrast with the present study, most extant research has not distinguished between overindebted and non-overindebted households and has not considered the possible differences (in terms of money attitudes, financial behaviors and their relation) between households who became overindebted due to internal (individualistic) versus external (situational) factors.

In the study here reported, we try to shed some light on these issues by assessing attitudes toward money of overindebted and non-overindebted consumers through its behavioral component (i.e., how much consumers take care and keep track of their money). Furthermore, consumers’ financial management behavior was assessed by means of a cash flow management scale (Godwin and Koonce, 1992) that includes three different categories of behaviors: record keeping, adjusting balance, and monitoring balance.

Our main goals are to evaluate (a) how money attitudes and money management behaviors differ across non-overindebted and (two types of) overindebted participants; and (b) how our measure of money attitudes is related to consumers’ money management behaviors. In short, we aim to address whether overindebted and non-overindebted individuals differ in a behavioral component of money attitudes, and whether this impacts financial management behavior above and beyond consumers’ indebtedness status.

## Methods

### Participants

Our sample consisted of 365 overindebted and non-overindebted participants. Overindebted participants were consumers who sought assistance and counseling with DECO (a Portuguese NGO for consumer defense) throughout 2017 regarding their problem with overindebtedness. Non-overindebted participants were collected through convenience sampling and asked if they were in a situation of overindebtedness, in which case they were recoded as overindebted (six participants were recoded this way, amounting to 236 overindebted and 129 non-overindebted consumers in the final sample). Fifty-eight participants identified as male and 76 as female, the remainder of the sample did not answer. Participants’ mean age was 50 years (SD = 15.58), the majority (73.94%) did not have a degree and lived alone (30.58%) or with another person (34.17%). Their household mean income per capita (i.e., total income divided by number of people in the household) was 796,93€ (SD = 654.67). Additional information is presented on Table 1.

**TABLE 1:** Socio-demographic characteristics of the two groups of overindebted and non-overindebted participants.

		Overindebted	Non-overindebted
**Age**			
	*M* (SD)	52.30 (11.66)	48.93 (17.61)
	Valid *N*	86	128
Income**			
	*M* (SD)	1,100.65 (562.54)	2,103.50 (2,176.07)
	Valid *N*	154	120
Income per capita**			
	*M* (SD)	597.87 (372.34)	1,042.84 (825.00)
	Valid *N*	147	119
Debt**			
	*M* (SD)	733.88 (944.51)	170.26 (222.74)
	Valid *N*	149	113
Debt to income ratio*			
	*M* (SD)	0.83 (1.89)	0.20 (0.17)
	Valid *N*	140	55
Debt + expenses to income ratio*			
	*M* (SD)	1.61 (2.19)	0.81 (0.50)
	Valid *N*	83	55
People in the household*			
	*M* (SD)	2.10 (1.00)	2.40 (1.27)
	Valid *N*	152	126
**Level of schooling**			
	1st Cycle	20 (12.82%)	3 (2.34%)
	2nd Cycle	13 (8.33%)	8 (6.25%)
	3rd Cycle	35 (22.43%)	26 (20.31%)
	Sec. Ed	59 (37.82%)	33 (25.78%)
	Voc. Ed	4 (2.56%)	9 (7.03%)
	Any degree	25 (16.02%)	49 (38.28%)
	Valid *N*	156	128
**Marital status**			
	Single	93 (59.23%)	74 (57.81%)
	(of whom divorced/separated)	45 (28.66%)	23 (17.96%)
	Married	64 (40.76%)	54 (42.18%)
	(of whom living together)	33 (21.01%)	54 (42.18%)
	Valid *N*	157	128
**Professional status**			
	Unemployed	31 (19.87%)	18 (14.4%)
	Informal jobs	3 (1.92%)	7 (5.6%)
	Retired	34 (21.79%)	41 (32.8%)
	Employed	88 (56.41%)	59 (47.2%)
	Valid *N*	156	125

*Difference between means is significant with p < 0.05. **Difference between means is significant with p < 0.001.

### Procedure

Overindebted participants responded to the questionnaire in either a paper and pencil format (those who went to DECO for a consultation) or in an editable computer file sent to them by mail (those who contacted DECO through their website or by email). Non-overindebted participants responded to the questionnaire in a paper and pencil format.

### Materials

Participants answered socio-demographic questions (marital status, level of schooling, professional status, number of people in the household) and questions concerning economic aspects of their life (monthly income, monthly expenses, and credit product installments). Reported causes of overindebtedness were collected by DECO and for some participants we were able to pair this information with our data (using codes that allowed to guarantee consumers anonymity). Participants identified one or more causes for their overindebtedness situation from several pre-defined options. These causes were categorized into two groups: ‘‘Internal causes’’ and ‘‘External causes.’’ The first included participants that identified ‘‘poor money management’’ and ‘‘excessive use of credit’’ as causes. The second included participants that identified ‘‘salary cuts,’’ ‘‘unemployment (self or spouse),’’ ‘‘divorce,’’ ‘‘death in the family,’’ and ‘‘birth in the family’’ (among other external causes), as causes for their overindebtedness. From the original 236 overindebted participants, 95 reported external causes and 36 reported internal causes for their overindebtedness. For the remaining 105 participants we could not identify the overindebtedness causes because participants did not report them or because we were not given access to this information due to confidentiality issues^1^.

Money attitudes were measured by asking participants to “Please indicate your degree of agreement with the following affirmations” referring to two items originally used by Lea et al. (1993), and obtaining the mean from both responses. The items used were “I am careless with money,” “I find it hard to keep track of my money” (Cronbach’s alpha 0.85). Participants responded to these items using a 5-point rating scale, from 1 (*Very strongly agree*) to 5 (*Very strongly disagree*). Money attitude was the mean between both responses. This measure was aimed at capturing the behavioral component of the multifaceted concept of money attitudes, which includes, in addition, a cognitive and an affective component (according to the tripartite model; Rosenberg and Hovland, 1960; Chatterjee et al., 2018).

Financial management behaviors were measured with 11 items taken from the Cash Flow Management scale described in Godwin and Koonce (1992) to which participants responded, using a 5-point scale from 1 (*Never*) to 5 (*Always*), to the question “Please indicate how often do you:” followed by the 11 items. A principal components analysis using the full sample yielded three components. The first component, with an eigenvalue of 5.56 and a Cronbach alpha of 0.89, was named “Record keeping” and includes the following financial behaviors: “Recording in writing most spending,” “Recording in writing your actual income,” “Assessing the amount of money spent on fixed expenses (rent, car, and payments, etc.),” “Assessing total expenses,” and “Assessing the amount of money spent on flexible expenses (food, clothing, and recreation, etc.).” The second component, with an eigenvalue of 1.26 and a Cronbach alpha of 0.70, was named “Adjusting balance,” and includes the following financial behaviors: “Thinking of ways to increase your income to match your needs” and “Try to think of ways to decrease your expenses to match your income.” The third component, with an eigenvalue of 1.02, and a Cronbach alpha of 0.78, was named “Monitoring balance” and includes the following behaviors: “Monitoring your spending to see if it is within your income,” “Monitoring your spending to see if it is in line with what you expected,”, Assessing the amount of money you can use during an emergency” and “Assessing the value of things you own.” Components values were the mean responses to their respective items. Money attitudes were measured prior to financial management behaviors. Finally, the questionnaire also included other measures collected for different research purposes, namely on the perceived causes of over-indebtedness causes, sleep quality, perceived health, feelings and locus of control, life satisfaction, financial satisfaction, well-being, and attitudes toward poverty.

## Results

Table 1 presents a comparative analysis of the main socio-demographic features of overindebted and non-overindebted consumers. Since overindebtedness is often related to low educational levels and income (Mandell, 1973; Chien and Devaney, 2001), and given that the two groups in our sample differed in education and income, in the data analyses presented below, we statistically controlled for the impact of these two variables on the relevant dependent measures (money attitudes and money management behaviors).

### Money Attitudes

A one-way analysis of covariance (ANCOVA) was performed with three levels of indebtedness status (overindebted as the result of internal causes; overindebted as the result of external causes; non-overindebted) as a between-participants factor and household income per capita and level of schooling as covariates. A main effect of indebtedness status, *F*(1, 198) = 16.54, *p* < 0.001, $\eta_{p}^{2}$ = 0.14, showed that non-overindebted participants have more positive attitudes toward money (i.e., a disposition to care and monitor their money; *M* = 4.29; SE = 0.29) than participants overindebted by external causes (*M* = 3.87; SE = 0.14), *F*(1, 198) = 2.38, *p* < 0.001, $\eta_{p}^{2}$ = 0.01, which in turn showed more positive attitudes than overindebted participants due to internal causes (*M* = 2.53, SE = 0.30), *F*(1, 198) = 1.92, *p* < 0.001, $\eta_{p}^{2}$ = 0.01. No other effects were significant. A similar result is found when the analysis is performed without the covariates, *F*(1, 203) = 18.32, *p* < 0.001, $\eta_{p}^{2}$ = 0.15.

In sum, non-overindebted consumers appear to show a positive attitude (as indicated by a mean response above the scale mid-point), consumers who become overindebted due to external factors showed weaker money attitudes (mean response close to the mid-point of the scale) and consumers who became overindebted due to internal factors showed the weakest money attitudes (below the mid-point of the scale).

### Financial Management Behaviors

We performed a 3 indebtedness status (overindebted as the result of internal causes; overindebted as the result of external causes; non-overindebted) × 3 financial management behaviors (record keeping, adjusting balance, and monitoring balance) mixed measures ANCOVA with the first factor between-participants, the second within participants and household income per capita and level of schooling as covariates.

The analysis yielded a main effect of financial behaviors, *F*(2, 400) = 14.26, *p* < 0.001, $\eta_{p}^{2}$ = 0.07. Adjusting balance was the more frequently reported behavior (*M* = 3.83, SE = 0.18), followed by monitoring balance (*M* = 3.16, SE = 0.18), and record keeping (*M* = 3.08, SE = 0.18). Post hoc comparisons (with a Bonferroni correction) revealed that while levels of monitoring balance and record keeping were not significantly different, both were significantly lower than adjusting balance (*p*s < 0.001).

There was also a significant interaction between overindebtedness status and financial management behaviors, *F*(4, 400) = 10.44, *p* < 0.001, $\eta_{p}^{2}$ = 0.09. Planned comparisons showed that this effect was driven by significant differences in monitoring balance, *F*(2, 200) = 12.29, *p* < 0.001, $\eta_{p}^{2}$ = 0.11, with non-overindebted participants reporting higher levels of monitoring balance (*M* = 3.81, SE = 0.09) than participants overindebted due to internal (*M* = 2.88, SE = 0.21), *F*(1, 200) = 2.02, *p* < 0.001, $\eta_{p}^{2}$ = 0.01 and external causes (*M* = 3.21, SE = 0.12), *F*(1, 200) = 1.94, *p* < 0.001, $\eta_{p}^{2}$ = 0.01. No other comparisons between conditions reached significance (all *p*s > 0.05).

**FIGURE 1:**
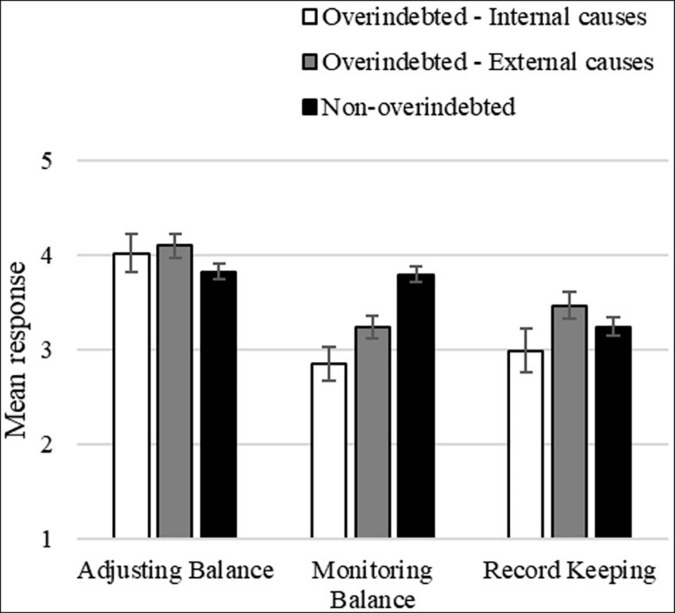
Mean reported frequency of financial management behaviors for participants overindebted as the result of internal and external causes and non-overindebted participants (1 – *Never*; 5 – *Always*).

Finally, there was a significant interaction between household income per capita and financial management behaviors, *F*(2, 400) = 3.25, *p* = 0.040, $\eta_{p}^{2}$ = 0.02, such that higher household income predicts less financial behaviors of adjusting balance (β = −0.145, *p* = 0.050). The same analysis without the covariates yields a similar result, with a significant main effect of financial behaviors, *F*(2, 430) = 56.03, *p* < 0.001, $\eta_{p}^{2}$ = 0.21, and a significant interaction between financial behaviors and overindebtedness status, *F*(4, 430) = *p* < 0.001, $\eta_{p}^{2}$ = 0.13 (Figure 1).

In sum, although the frequency with which consumers adopted different kinds of financial management behaviors varies, we found no differences in the reported frequency of financial behaviors between consumers overindebted due to external and internal causes, and non-overindebted consumers, except for the monitoring balance behaviors. Overindebted consumers, regardless of the reported causes, engage significantly less in these kinds of behaviors than non-overindebted consumers. Therefore, monitoring balance behaviors (i.e., continuous monitoring of actual and future expenses in relation to one’s own income or wealth) appears to be associated with keeping expenses and debt service within manageable levels.

### Relationship Between Money Attitudes and Financial Management Behaviors

In line with previous analyses, zero-order correlations (see Table 2) show that indebtedness status (not being overindebted was coded 0 and being overindebted was coded 1) is associated with weaker money attitudes and poor monitoring balance. Naturally, overindebted consumers are also associated with larger debt-to-income ratios. Money attitudes are positively correlated with all measures of financial behaviors. In other words, higher levels of money attitudes are associated with better money management. The three measures of financial management behaviors are positively correlated with each other. Finally, money attitudes are negatively associated to debt-to-income ratio.

**TABLE 2:** Correlations between overindebtedness status, money attitudes, financial management behaviors, and debt-to-income ratio (*N* = 152).

	Mean/proportion	SD	2	3	4	5	6
1 – Overindebtedness status (1 = overindebted)	0.64	0.48	0.47**	−0.37**	0.01	0.02	−0.36**
2 – Debt-to-income ratio	0.48	0.45		−0.17*	0.06	0.07	–0.10
3 – Money attitude	3.91	1.17			0.40**	0.36**	0.55**
4 – Record keeping	3.42	1.08				0.57**	0.63**
5 – Adjusting balance	4.06	0.90					0.55**
6 – Monitoring balance	3.47	0.97					

*p < 0.05; **p < 0.001.

To further explore the relationship between our measure of the behavioral component of money attitudes and financial management behaviors, a multiple regression analysis was conducted to test the predictive role of overindebtedness and money attitudes (i.e., a disposition to care and monitor their money) on record keeping, adjusting balance, and monitoring balance behaviors, controlling for household income per capita and education level, using *MPlus* 7.2 (Muthén and Muthén, 1998–2012). Based on the results of the previous analyses, significant correlations between predictors and between criterion variables were included in the model. To evaluate model fit, the following indexes and criteria were used: the comparative fit index (CFI) above 0.95, the root mean square error of approximation (RMSEA), and the standardized root mean residual (SRMR) below 0.08, all indicative of a good fit (Hu and Bentler, 1999; Kline, 2011).

**FIGURE 2:**
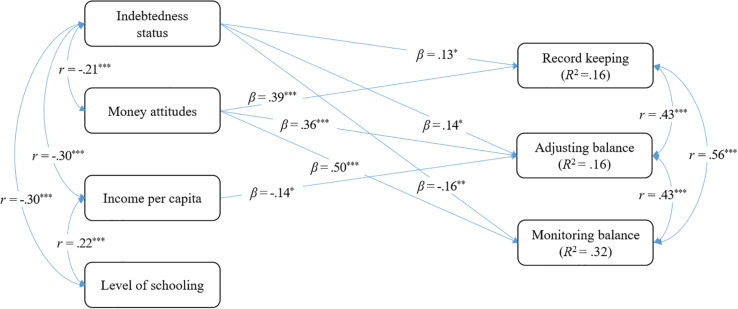
Model examining the predictive role of overindebtedness and money attitudes on financial management behaviors. *p < 0.05; **p < 0.01; ***p < 0.001.

The multiple regression model fit the data well: χ^2^(2) = 2.03, *p* = 0.36; CFI = 1.00; RMSEA = 0.01, 90% CI: 0.00, 0.10; SRMR = 0.02. As depicted in Figure 2, results revealed that overindebtedness and money attitudes are negatively associated with each other, but both significantly predicted all three financial management behaviors analyzed. More specifically, both overindebtedness and money attitudes were associated with higher levels of record keeping (*B* = 0.30, *p* = 0.023 / β = 0.13, *p* = 0.022 and *B* = 0.37, *p* < 0.001 / β = 0.39, *p* < 0.001, respectively), and adjusting balance (*B* = 0.26, *p* = 0.019 / β = 0.14, *p* = 0.018 and *B* = 0.29, *p* < 0.001 / β = 0.36, *p* < 0.001, respectively). However, while money attitudes were also associated with higher levels of monitoring balance (*B* = 0.43, *p* < 0.001 / β = 0.50, *p* < 0.001), overindebtedness was associated with lower levels of this financial management behavior (*B* = −32, *p* = 0.003 / β = −0.16, *p* = 0.002). Regarding the covariates, household income per capita was associated with lower levels of adjusting balance behavior, while education level did not predict any of the financial management behaviors. The whole model, respectively, explained 15.5, 16.2, and 31.5% of the variance of record keeping, adjusting balance, and monitoring balance.

Next, to account for the different causes of overindebtedness, we once more divided the sample in three groups – (1) overindebted due to internal causes, (2) overindebted due to external causes, and (3) non-overindebted participants – and performed a multiple group analysis with AMOS (v. 26) to test if the model significantly differed between the three groups. An unconstrained multiple group model, with all paths allowed to be freely estimated across the three groups was compared to a model where all paths were constrained to be equivalent across groups. Results of this analysis revealed a non-significant chi-square difference between the unconstrained and the constrained models: Δχ^2^(18) = 23.36, *p* = 0.177, indicating that the model does not vary significantly between the three groups^2^. Descriptive statistics (means and standard deviations) of money attitudes and financial management behaviors by group are presented in Table 1 of the Supplementary Materials.

In addition, despite the high percentage of missing data on participants’ gender (63.3%), we analyzed the potential moderating role of gender in the proposed model, by performing another multi-group analysis with the sample for which this data was available, using AMOS (v. 26). An unconstrained multi-group model, with all paths allowed to be freely estimated for both men and women was compared to a model where all paths were constrained to be equivalent for both groups. Results of this analysis also showed a non-significant chi-square difference between the unconstrained and the constrained models: Δχ^2^(9) = 7.86, *p* = 0.549, indicating that the model does not vary significantly between men and women. Descriptive statistics (means and standard deviations) of money attitudes and financial management behaviors by gender are presented in Table 2 of the Supplementary Materials.

At last, we also analyzed the potential moderating role of participants’ age in associations between money attitudes and the three financial management behaviors, using the PROCESS macro (v. 3) for SPSS Model 1 (Hayes, 2018), controlling for indebtedness status, household income per capita, and education level. Results did not reveal a significant interaction between participants’ age and money attitudes. Thus, age was not a moderator of the hypothesized associations.

## Discussion

This paper explored whether overindebted and non-overindebted consumers differ in a measure of money attitudes (the disposition to care for and monitor their money) and three types of financial management behaviors. Our findings indicate that non-overindebted consumers display stronger money attitudes than overindebted consumers. Moreover, consumers who became overindebted due to internal causes showed weaker attitudes (i.e., weaker dispositions to care/monitor their money) than consumers who became overindebted due to external causes. This suggests two ways by which overindebtedness may be related to our measure of attitudes toward money. In the case of overindebtedness due to internal causes, weak money attitudes are likely to work as a risk factor, whereas the relative weaker attitudes of consumers who became overindebted due to external factors (compared to non-overindebted consumers) are more likely to be a consequence of their life circumstances. We tried to find empirical support for the above by performing a multiple group analysis with the same three subgroups but the analysis revealed no significant differences. However, the fact that the reported causes of over indebtedness were missing for about 44% of the overindebted consumers greatly reduced the sample size we could use for this analysis and thus the ability to statistically discriminate between the subgroups. Future research with a larger sample (and hopefully less missing data) is necessary to clarify this point.

In terms of financial management behaviors, overindebted consumers reported monitoring their balance (of expenses versus income) more seldom than non-overindebted consumers. In contrast, both groups report engaging in the remaining financial behaviors (record keeping and adjusting balance) with the same frequency. These two money management behaviors appear, thus, to be poor candidates to distinguish one group from the other.

We also explored the impact of indebtedness status and money attitude on the three categories of financial management behaviors (record keeping, adjusting balance, and monitoring balance) in a regression analysis, while controlling for income and education. Overindebtedness was positively associated with record keeping and adjusting balance behaviors while negatively associated with money attitude. In other words, overindebted consumers (compared to their non-overindebted counterparts) show a stronger tendency to record their fixed (e.g., rent payments) and flexible expenses (e.g., clothing, recreation) in writing, as well as their actual income. Overindebted consumers also engage more often in thinking of ways to increase their income (to match their needs) and decrease their expenses (to match their income). In contrast, they engage in less monitoring of their actual expenses (i.e., checking if they were in line with what was expected), they less often assess the money they could use during an emergency as well as the value of the things they own. The financial difficulties stemming from overindebtedness may be seen as creating the need for more systematic behaviors of record keeping and adjusting balance while reducing consumers’ ability and/or opportunity to engage in monitoring balance. On the other hand, consumers’ attitudes toward money appears to increase their engagement in all three categories of money management behaviors, above and beyond their indebtedness status.

In sum, overindebtedness and money attitudes are both positively associated with record keeping and adjusting balance behaviors. Perhaps consumers engage in more of these two types of financial behaviors either due to the fact that they become overindebted, that they have stronger money attitudes (i.e., disposition to care for their money), or both. In contrast, overindebtedness and money attitudes have opposite effects on monitoring balance: overindebted consumers and consumers with weaker money attitudes show fewer monitoring balance behaviors. This type of money management behavior is more oriented to preventing future financial difficulties as they focus on comparing current and expected expenses, money to be used in emergencies, and on evaluating one’s belongings (which may be seen as a way of obtaining cash, if need be). While consumers with weaker money attitudes may engage less in these types of behaviors due to feebler dispositions to care for their money, overindebted consumers may do so as the result of their difficult financial circumstances. Indeed, the expenses of overindebted consumers are more likely to be systematically above what they should be, they are seldom able to consider saving money for emergencies, and their belongings with financial value are often pledged. Lower levels of account balance monitoring displayed by overindebted consumers could reflect higher present bias, which has been documented in individuals facing financial difficulties, and hinders their capacity to improve their situation (Mullainathan and Shafir, 2013).

Taken together, the results of this study allow the following conclusions to be drawn: (a) our measure of money attitudes and monitoring balance discriminate between overindebted and non-overindebted consumers; and (b) non-overindebted consumers and consumers with stronger money attitudes (regardless of whether they are overindebted or not) tend to more often engage in monitoring balance.

Almenberg et al. (2018) showed that debt attitude, more specifically discomfort with debt, was associated with lower debt levels. They suggested that such discomfort may act as a self-imposed borrowing constraint stemming from social norms. Our study adds to this previous result by showing an association between money attitude and consumers debt. Consumers with weaker money attitudes tended to have higher debt to income ratios [one of the debt indicators also used by Almenberg et al. (2018)].

In addition, the negative association that emerged in the present study between money attitudes and indebtedness status – non-overindebted consumers tend to show stronger money attitudes than overindebted ones – suggests that lower levels of positive attitudes toward money may work as a relevant risk factor of overindebtedness.

### Limitations and Social Implications

Money attitudes have a multifaceted nature with different dimensions (e.g., Yamauchi and Templer, 1982), which according to the tripartite model of attitudes (Rosenberg and Hovland, 1960; Chatterjee et al., 2018), may be classified in three components (or indicators): a cognitive (e.g., money as achievement), an affective (money as a source of anxiety), and a behavioral component (budget and money monitoring). However, research using different measures of money attitudes, such as Yamauchi and Templer’s (1982) MAS, and Furnham’s (1984) Money Beliefs and Behavior Scale, often ignore these instruments multifactorial dimensions and use a money attitude index across all (or part of the) scales items (e.g., Qamar et al., 2016; Sabri et al., 2020). In our case, the money attitude measure only tapped the behavioral component of this attitude and included only two items. Although single-item measures have been used before to assess similar concepts (e.g., Almenberg et al., 2018 measured attitudes toward debt by asking one simple question “do you feel uncomfortable with having debt?”), these are important limitations of the present study that should be addressed in future research by using multidimensional measures to assess the three components of the money attitudes with multiple items per component. We speculate that the affective component of money attitudes, in particular, is likely to play an important role given that overindebtedness is often associated with depression, anxiety, and social stigma.

The correlational nature of the study reported herein, does not make it possible to ascertain causality. The causality issue is particularly troublesome for the regression analyses conducted to explore the relationship between our measure of money attitudes and financial management behaviors. Even so, given the more abstract nature of the attitude items and the more specific and concrete nature of the financial behavior items, having the former predicting the latter is in line with a considerable amount of literature on attitude-behavior consistency, according to which general predispositions or attitudes are used to predict behavior (e.g., Maio and Haddock, 2004; Fishbein and Ajzen, 2010). In other words, it appears to be theoretically sounder to consider and test the hypothesis that money attitudes predict engagement in specific money management behaviors, than the inverse: a specific financial behavior being the cause of a more general money-related attitude. Nevertheless, we acknowledge that it is not possible in the present study to fully disentangle the two causal directions. According to the self-determination theory (e.g., Bem, 1972), it might be the case that at least some consumers used their own financial behavior to infer their dispositions toward money (even if the money attitude measure was assessed before the financial management behaviors in our questionnaire). Regardless of which causal direction is dominant or stronger, the association between money attitudes and the three types of financial management behaviors suggests that interventions to improve one of them could have beneficial effects on the other.

Future research could use longitudinal designs (with at least two data collection waves) to measure the impact of debt-related attitudes on money management behavioral tasks, to more clearly ascertain causality.

Given the self-reported nature of our measures, response social desirability may have biased our results to a certain extent. Future research should thus replicate our findings using a paradigm more robust to social desirability bias, which can achieve considerable lengths in surveys on sensitive topics (Tourangeau and Yan, 2007; Gittelman et al., 2015). This could be achieved via indirect or implicit indicators of attitudes and behaviors or more easily by including a scale to account for individual differences in social desirability responding, so that its effect can be statistically controlled for.

Finally, although we controlled for differences in level of education and income (and tested for the impact of age and gender in the regression model) between overindebted and non-overindebted consumers in our sample, it is always possible that the two groups differ in yet other variables (besides the indebtedness status). These other differences may have contributed to explaining the reported findings. In the same vein, as participants completed other questionnaires before responding to the measures used in the present paper, their influence on these measures cannot be excluded.

## Conclusion

Standard theories of consumption such as the life cycle hypothesis assume that people plan ahead taking on debt based on expected future income when they are young and then save during middle age to maintain consumption level later in life (Modigliani, 1966). In practice many consumers seem to deviate for these theoretical predictions when it comes to borrowing and saving. Factors that have been called upon to explain these discrepancies include variations in self-regulation and ego-depletion across individuals (Baumeister and Vohs, 2007; Mullainathan and Shafir, 2013), financial illiteracy (e.g., Lusardi, 2012) and reasoning biases (e.g., Thaler and Sunstein, 2008). In this paper, we provide some evidence concerning the importance of an additional factor, money attitudes (here defined as consumers behavioral disposition to care and monitor their money). Specifically, the impact of money attitudes in consumers money management and borrowing behavior suggests that education programs that focus on reducing financial illiteracy (e.g., Lusardi, 2019) or heuristic-based errors and biases (e.g., Soll et al., 2013) may be beneficially complemented by interventions that could directly foster (a) social norms to care and monitoring one’s finances (see Moffitt, 2001); and (b) financial behaviors of monitoring balance (e.g., coaching people to anticipate and be prepared for financial emergencies).

## Data Availability Statement

The raw data supporting the conclusions of this article will be made available by the authors, without undue reservation.

## Ethics Statement

The studies involving human participants were reviewed and approved by Ethics committee of the Faculty of Psychology, University of Lisbon. The participants provided their written informed consent to participate in this study.

## Author Contributions

FA, MF, and JS contributed equally to conceptualization, data collection, analysis, and manuscript writing. CS provided indispensable analysis and inputs on results and discussion. All authors read and approved the final version of this article.

## Conflict of Interest

The authors declare that the research was conducted in the absence of any commercial or financial relationships that could be construed as a potential conflict of interest.

## Footnotes

1 Because of this substantial increase in the missing data for the sample included in the analysis of money attitudes and financial management behaviors, we also performed ANCOVAs ignoring causes of overindebtedness, with the sample simply divided in overindebted and non-overindebted participants. These analyses and their results are described in the Supplementary Material. The pattern of results is similar to the one found when the causes of overindebtedness are taken into account.

2 Results of the multi-group analysis considering only the two groups of overindebted participants also showed that the model did not significantly differ between these groups, Δχ^2^(9) = 14.88, *p* = 0.094. That is, there is no moderating effect of cause of overindebtedness.

## References

[B1] Almenberg J. Lusardi A. äve-Söderbergh J. S. Vestman J. R. (2018). *Attitudes Toward Debt and Debt Behavior (NBER Working Paper No. 24935).* Cambridge, MA: National Bureau of Economic Research, Inc. 10.3386/w24935

[B2] Ameriks J. Caplin A. Leahy J. (2003). Wealth accumulation and the propensity to plan. *Q. J. Econ.* 118 1007–1047. 10.1162/00335530360698487

[B3] Ameriks J. Caplin A. Leahy J. Tyler T. (2007). Measuring self-control problems. *Am. Econ. Rev.* 97 966–972. 10.1257/aer.97.3.966

[B4] Ando A. Modigliani F. (1963). The “life cycle” hypothesis of saving: aggregate implications and tests. *Am. Econ. Rev.* 53 55–84. 10.4236/eng.2016.83009

[B5] Baumeister R. F. Vohs K. D. (2007). Self-regulation, ego depletion, and motivation. *Soc. Personal. Psychol. Compass* 1 115–128. 10.1111/j.1751-9004.2007.00001.x

[B6] Baumeister R. F. Vohs K. D. Tice D. M. (2007). The strength model of self-control. *Curr. Dir. Psychol. Sci.* 16 351–355. 10.1111/j.1467-8721.2007.00534.x

[B7] Bem D. J. (1972). “Self-perception theory,” in *Advances in Experimental Social Psychology*, Vol. 6 ed. Berkowitz L. (New York, NY: Academic Press), 1–62. 10.1016/S0065-2601(08)60024-6

[B8] Bloom B. L. Niles R. L. Tatcher A. M. (1985). Sources of marital dissatisfaction among newly separated persons. *J. Fam. Issues* 6 359–373. 10.1177/019251385006003007

[B9] Bover O. Casado J. M. Costa S. du Caju P. McCarthy Y. Sierminska E. (2016). The distribution of debt across euro area countries: the role of individual characteristics, institutions and credit conditions. *Int. J. Cent. Bank.* 12 71–128.

[B10] Canner G. B. Luckett C. A. Cook W. C. Middleton N. D. (1991). Payment of household debts. *Fed. Res. Bull.* 77 218–229.

[B11] Cecchetti S. G. Mohanty M. Zampolli F. (2011). “Achieving growth amid fiscal imbalances: the real effects of debt,” in *Proceedings of the Economic Symposium*, Vol. 352 (Kansas City, MO: Federal Reserve Bank of Kansas City), 145–196.

[B12] Chatterjee D. Keswani T. Gupta S. (2018). *Money Attitudes of Indian Adults: An Exploratory Study (Working paper, March 2, 2018).* 10.2139/ssrn.3683299

[B13] Chien Y. Devaney S. (2001). The effects of credit attitude and socioeconomic factors on credit card and installment debt. *J. Consum. Aff.* 35 162–179. 10.1111/j.1745-6606.2001.tb00107.x

[B14] D’Alessio G. Iezzi S. (2013). *Household Over-Indebtedness: Definition and Measurement with Italian Data. Questioni di Economia e Finanza (Occasional Paper)*, Vol. 149. Rome: Bank of Italy, 1–26. 10.2139/ssrn.2243578

[B15] Dew J. (2011). The association between consumer debt and the likelihood of divorce. *J. Fam. Econ. Issues* 32 554–565. 10.1007/s10834-011-9274-z

[B16] Donnelly G. Iyer R. Howell R. T. (2012). The big five personality traits, material values, and financial well-being of self-described money managers. *J. Econ. Psychol.* 33 1129–1142. 10.1016/j.joep.2012.08.00

[B17] Dowling N. A. Corney T. Hoiles L. (2009). Financial management practices and money attitudes as determinants of financial problems and dissatisfaction in young male Australian workers. *J. Financ. Couns. Plan.* 20 5–13.

[B18] Eagly A. H. Chaiken S. (1993). *The Psychology of Attitudes.* Fort Worth, TX: Harcourt Brace Jovanovich College Publishers.

[B19] Fan J. Chang Y. Hanna S. (1993). Real income growth and optimal credit use. *Financ. Serv. Rev.* 3 45–58. 10.1016/1057-0810(93)90005-B

[B20] Ferreira M. B. Pinto D. C. Herter M. M. Soro J. C. Vanneschi L. Castelli M. (2020). Using artificial intelligence to overcome over-indebtedness and fight poverty. *J. Bus. Res.* 10.1016/j.jbusres.2020.10.035 33100428 PMC7571461

[B21] Fishbein M. Ajzen I. (2010). *Predicting and Changing Behavior: The Reasoned Action Approach.* New York, NY: Psychology Press.

[B22] Furnham A. (1984). Money sides of the coin: the psychology of money usage. *Pers. Individ. Differ.* 5 501–509. 10.1016/0191-8869(84)90025-4

[B23] Gittelman S. Lange V. Cook W. A. Frede S. M. Lavrakas P. J. Pierce C. (2015). Accounting for social-desirability bias in survey sampling: a model for predicting and calibrating the direction and magnitude of social-desirability bias. *J. Advert. Res.* 55 242–254. 10.2501/JAR-2015-006

[B24] Godwin D. Koonce J. (1992). Cash flow management of low-income newlyweds. *Financ. Couns. Plan.* 3 17–42.

[B25] Haas O. J. (2006). *Overindebtedness in Germany (International Labour Organization Working Paper No. 44).* Available online at: https://www.ilo.org/wcmsp5/groups/public/—ed_emp/documents/publication/wcms_117963.pdf (accessed April 2, 2021).

[B26] Hanna S. Fan J. Chang Y. (1995). Optimal life cycle savings. *Financ. Couns. Plan.* 6 1–15.

[B27] Hayes A. F. (2018). *Introduction to Mediation, Moderation, and Conditional Process Analysis*, 2nd Edn. New York, NY: The Guilford Press.

[B28] Hayhoe C. R. Leach L. Turner P. R. (1999). Discriminating the number of credit cards held by college students using credit and money attitudes. *J. Econ. Psychol.* 20 643–656. 10.1016/S0167-4870(99)00028-8

[B29] Hu L. T. Bentler P. M. (1999). Cutoff criteria for fit indexes in covariance structure analysis: conventional criteria versus new alternatives. *Struct. Equ. Modeling* 6 1–55. 10.1080/10705519909540118

[B30] Kahler R. Fox K. (2005). *Conscious Finance: Uncover Your Hidden Money Beliefs and Transform the Role of Money in Your Life.* Rapid City: FoxCraft.

[B31] Kaiser F. G. Wilson M. (2019). The Campbell paradigm as a behavior-predictive reinterpretation of the classical tripartite model of attitudes. *Eur. Psychol.* 24 359–374. 10.1027/1016-9040/a000364 32116425 PMC7039345

[B32] Kerkmann B. C. Lee T. R. Lown J. M. Allgood S. M. (2000). Financial management, financial problems and marital satisfaction among recently married university students. *J. Financ. Couns. Plan.* 11 55–65.

[B33] Kline R. (2011). *Principles and Practice of Structural Equation Modelling*, 3rd. Edn. New York, NY: Guilford Press.

[B34] Klontz B. T. Britt S. L. (2012). How clients’ money scripts predict their financial behaviors. *J. Financial Plan.* 25 33–43. https://growcounseling.com/wp-content/uploads/2018/03/Money-Scripts-Assessment.pdf

[B35] Klontz B. Britt S. L. Mentzer J. Klontz T. (2011). Money beliefs and financial behaviors: development of the Klontz money script inventory. *J. Financ. Ther.* 2:1. 10.4148/jft.v2i1.451

[B36] Ksendzova M. Donnelly G. Howell R. (2017). A brief money management scale and its associations with personality, financial health, and hypothetical debt repayment. *J. Financ. Couns. Plan.* 28 62–75.

[B37] Lay A. Furnham A. (2019). A new money attitudes questionnaire. *Eur. J. Psychol. Assess.* 35 813–822. 10.1027/1015-5759/a000474

[B38] Lea S. E. Webley P. Levine R. M. (1993). The economic psychology of consumer debt. *J. Econ. Psychol.* 14 85–119. 10.1016/0167-4870(93)90041-I

[B39] Lea S. E. Webley P. Walker C. M. (1995). Psychological factors in consumer debt: money management, economic socialization, and credit use. *J. Econ. Psychol.* 16 681–701. 10.1016/0167-4870(95)00013-4

[B40] Lusardi A. (2012). Numeracy, financial literacy, and financial decision-making. *Numeracy* 5:2. 10.5038/1936-4660.5.1.2

[B41] Lusardi A. (2019). Financial literacy and the need for financial education: evidence and implications. *Swiss J. Econ. Stat.* 155 1–8. 10.1186/s41937-019-0027-5

[B42] Lusardi A. Mitchell O. S. Oggero N. (2018). “The changing face of debt and financial fragility at older ages,” in *Proceedings of the AEA Papers and Proceedings*, Vol. 108 (Nashville, TN: American Economic Association), 407–411. 10.1257/pandp.20181117

[B43] Lusardi A. Tufano P. (2015). Debt literacy, financial experiences, and overindebtedness. *J. Pension Econ. Financ.* 14 332–368. 10.3386/w14808

[B44] Maio G. R. Haddock G. (2004). “Theories of attitude: creating a witches’ brew,” in *Contemporary Perspectives on the Psychology of Attitudes*, eds Haddock G. Maio G. R. (Hove: Psychology Press), 425–453.

[B45] Mandell L. (1973). Consumer knowledge and understanding of consumer credit. *J. Consum. Aff.* 7 23–36. 10.1111/j.1745-6606.1973.tb00518.x

[B46] Mani A. Mullainathan S. Shafir E. Zhao J. (2013). Poverty impedes cognitive function. *Science* 341 976–980. 10.1126/science.1238041 23990553

[B47] Modigliani F. (1966). The life cycle hypothesis of saving, the demand for wealth and the supply of capital. *Soc. Res.* 33 160–217.

[B48] Moffitt R. A. (2001). “Policy interventions, low-level equilibria, and social interactions,” in *Social Dynamics*, eds Durlauf S. N. Young H. P. (Cambridge, MA: MIT Press), 45–82.

[B49] Mullainathan S. Shafir E. (2013). *Scarcity: Why Having Too Little Means So Much.* New York, NY: Henry Holt & Company.

[B50] Muthén L. K. Muthén B. O. (1998–2012). *Mplus User’s Guide*, 7th Edn. Los Angeles, CA: Muthén & Muthén.

[B51] Niemi-Kiesiläinen J. (2009). “Over-indebted households and law: prevention and rehabilitation in Europe,” in *Consumer Credit, Debt and Bankruptcy: Comparative and International Perspectives*, eds Niemi J. Ramsey I. Whitford W. C. (Oxford: Hart Publishing), 91–104.

[B52] Norvilitis J. Merwin M. Osberg T. Roehling P. Young P. Kamas M. (2006). Personality factors, money attitudes, financial knowledge, and credit-card debt in college students. *J. Appl. Soc. Psychol.* 36 1395–1413. 10.1111/j.0021-9029.2006.00065.x

[B53] Norvilitis J. Szablicki P. Wilson S. (2003). Factors influencing levels of credit-card debt in college Students. *J. Appl. Soc. Psychol.* 33 935–947. 10.1111/j.1559-1816.2003.tb01932.x

[B54] O’Neill B. Prawitz A. Sorhaindo B. Kim J. Garman E. T. (2006). Changes in health, negative financial events, and financial distress/financial well-being for debt management program clients. *J. Financ. Couns. Plan.* 17 46–63.

[B55] Peltier J. W. Dahl A. J. Schibrowsky J. E. (2016). Sequential loss of self-control: exploring the antecedents and consequences of student credit card debt. *J. Financ. Serv. Mark.* 21 167–181. 10.1057/s41264-016-0002-5

[B56] Qamar M. A. J. Khemta M. A. N. Jamil H. (2016). How knowledge and financial self-efficacy moderate the relationship between money attitudes and personal financial management behavior. *Eur. Online J. Nat. Soc. Sci.* 5:296.

[B57] Robb C. (2011). Financial knowledge and credit card behavior of college students. *J. Fam. Econ. Issues* 32 690–698. 10.1007/s10834-011-9255-2

[B58] Robb C. Pinto M. (2010). College students and credit card use: an analysis of financially at-risk students. *Coll. Stud. J.* 44 823–835.

[B59] Roberts J. Sepulveda C. (1999). Money attitudes and compulsion buying. *J. Int. Consum. Mark.* 11 53–79. 10.1300/J046v11n04_04

[B60] Rosenberg M. J. Hovland C. I. (1960). “Cognitive, affective, and behavioral components of attitudes,” in *Attitude Organization and Change*, eds Rosenberg M. Hovland C. McGuire W. Abelson R. Brehm J. (New Haven, CT: Yale University Press), 1–14.

[B61] Sabri M. Wijekoon R. Rahim H. (2020). The influence of money attitude, financial practices, self-efficacy and emotion coping on employees’ financial well-being. *Manag. Sci. Lett.* 10 889–900. 10.5267/j.msl.2019.10.007

[B62] Soll J. B. Keeney R. L. Larrick R. P. (2013). Consumer misunderstanding of credit card use, payments, and debt: causes and solutions. *J. Public Policy Mark.* 32 66–81. 10.1509/jppm.11.061 11670861

[B63] Tang T. L. P. (1992). The meaning of money revisited. *J. Organ. Behav.* 13 197–202. 10.1002/job.4030130209

[B64] Tang T. L. P. (1993). The meaning of money: extension and exploration of the money ethic scale in a sample of university students in Taiwan. *J. Organ. Behav.* 14 93–99. 10.1002/JOB.4030140109

[B65] Tang T. L. P. (1995). The development of a short money ethic scale: attitudes toward money and pay satisfaction revisited. *Pers. Individ. Differ.* 19 809–816. 10.1016/S0191-8869(95)00133-6

[B66] Thaler R. Sunstein C. (2008). *Nudge Theory.* London: Penguin Books.

[B67] Tourangeau R. Yan T. (2007). Sensitive questions in surveys. *Psychol. Bull.* 133 859–883. 10.1037/0033-2909.133.5.859 17723033

[B68] Xiao J. J. Sorhaindo B. Garman E. T. (2006). Financial behaviours of consumers in credit counselling. *Int. J. Consum. Stud.* 30 108–121. 10.1111/j.1470-6431.2005.00455.x

[B69] Yamauchi K. T. Templer D. J. (1982). The development of a money attitude scale. *J. Pers. Assess.* 46 522–528. 10.1207/s15327752jpa4605_14 16367635

