# Supplementary material for: Attitudes Toward Money and Control Strategies of Financial Behavior: A Comparison Between Overindebted and Non-overindebted Consumers
Source: Front Psychol. 2021 Apr 16;12:566594. doi: 10.3389/fpsyg.2021.566594 (PMC8085487; doi:10.3389/fpsyg.2021.566594)
Supplement: Supplementary file 1 [file Table_1.DOCX]

Supplementary Material

# Supplementary Analyses

**Money Attitudes by Indebtedness Status (overindebted vs. non-overindebted)**

Here we report on results of a one-way ANCOVA was performed with indebtedness status: overindebted, non-overindebted as a between-participants factor and household income per capita and level of schooling as covariates. The analysis showed that non-overindebted participants have more positive money attitudes (*M* = 4.32; *SE* = .10) than overindebted participants (*M* = 3.62; *SE* = .11, *F*(1, 219) = 20.10, *p* < .001, *η_p_^2^* = .08. No other effects were significant. A similar result is found when the analysis is performed without the covariates, *F*(1, 306) = 19.66, *p* < .001, *η_p_^2^* = .06.

In sum, although both groups of consumers appear to have positive money attitudes (both response means are above the scale mid-point), overindebted consumers are less financially accountable than non-overindebted consumers.

**Financial Management Behaviors by Indebtedness Status (overindebted vs. non-overindebted)**

Here we report on results of a 2 (indebtedness status: overindebted, non-overindebted) x 3 (financial management behaviors: record keeping, adjusting balance, and monitoring balance) mixed measures ANCOVA with the first factor between-participants, the second within participants, and household income per capita and level of schooling as covariates.

The analysis yielded a main effect of financial behaviors, *F*(2, 440) = 8.55, *p* < .001, *η_p_^2^* = .04. More specifically, different financial management behaviors varied in frequency. Adjusting balance was the more frequently reported behavior (*M* = 3.95, *SE* = 0.06), followed by monitoring balance (*M* = 3.50. *SE* = 0.06), and record keeping (*M* = 3.31, *SE* = 0.07). Post hoc comparisons (with a Bonferroni correction) revealed that all differences between measures of financial behaviors were significant (all *p*s < .001).

There was also a significant interaction between overindebtedness status and financial management behaviors, *F*(2, 440) = 19.14, *p* < .001, *η_p_^2^* = .08. Planned comparisons showed that this effect was driven by significant differences in monitoring balance, *F*(1, 220) = 21.72, *p* < .001, *η_p_^2^* = .09, with overindebted participants reporting lower levels of monitoring balance (M = 3.19, SE = 0.10) than non-overindebted participants (*M* = 3.82, *SE* = 0.09). No other comparisons between conditions reached significance (all *F*s < 1).

Finally, there was a significant interaction between household income per capita and financial management behaviors, *F*(2, 440) = 3.79, *p* = .023, *η_p_^2^* = .02, such that higher household income predicts less financial behaviors of adjusting balance (β = - .144, p = .043). The same analysis without the covariates yields a similar result, with a significant main effect of financial behaviors, *F*(2, 616) = 72.94, *p* < .001, *η_p_^2^* = .19, and a significant interaction between financial behaviors and overindebtedness status, *F*(2, 616) = *p* < .001, *η_p_^2^* = .09.

In sum, although the frequency with which consumers adopted different kinds of financial management behaviors varies, we found no differences in the reported frequency of financial behaviors between overindebted and non-overindebted consumers, except for the monitoring balance behaviors. Overindebted consumers engage significantly less in these kinds of behaviors than non-overindebted consumers. Therefore, monitoring balance behaviors (i.e., continuous monitoring of actual and future expenses in relation to one’s own income or wealth) appears to be associated with keeping expenses and debt service within manageable levels.

# Supplementary Tables

Table S1.

Descriptive statistics by group

|  | Group | *M* | *SD* |
| --- | --- | --- | --- |
| Money Attitude | OI – lack of acquired skills | 2.83 | 1.33 |
|  | OI – unexpected life events | 3.85 | 1.11 |
|  | NOI | 4.28 | 0.94 |
| Record keeping | OI – lack of acquired skills | 2.99 | 1.04 |
|  | OI – unexpected life events | 3.48 | 1.14 |
|  | NOI | 3.26 | 1.06 |
| Adjusting balance sheet | OI – lack of acquired skills | 4.07 | 0.94 |
|  | OI – unexpected life events | 4.07 | 1.01 |
|  | NOI | 3.84 | 0.87 |
| Monitoring balance sheet | OI – lack of acquired skills | 2.82 | 0.82 |
|  | OI – unexpected life events | 3.22 | 1.03 |
|  | NOI | 3.80 | 0.92 |
| *Note*. *M* = Mean; *SD* = Standard Deviation; OI = Overindebted; NOI = Non-overindebted | | | |

Table S2.

Descriptive statistics by gender

|  | Gender | *M* | *SD* |
| --- | --- | --- | --- |
| Money attitude | Female | 4.18 | 1.09 |
|  | Male | 4.31 | 0.79 |
| Record keeping | Female | 3.31 | 1.13 |
|  | Male | 3.09 | 0.95 |
| Adjusting balance sheet | Female | 3.84 | 0.84 |
|  | Male | 3.78 | 0.88 |
| Monitoring balance sheet | Female | 3.73 | 0.96 |
|  | Male | 3.75 | 0.90 |

*Note. M* = Mean; *SD* = Standard deviation.
